# Erythropoietin Attenuates Postoperative Cognitive Dysfunction by Shifting Macrophage Activation toward the M2 Phenotype

**DOI:** 10.3389/fphar.2017.00839

**Published:** 2017-11-16

**Authors:** Jae Hoon Lee, Eun Hee Kam, So Yeon Kim, So Yeong Cheon, Eun Jung Kim, Seungsoo Chung, Ji-Hyun Jeong, Bon-Nyeo Koo

**Affiliations:** ^1^Department of Anesthesiology and Pain Medicine, Severance Hospital, Seoul, South Korea; ^2^Anesthesia and Pain Research Institute, College of Medicine, Yonsei University, Seoul, South Korea; ^3^Department of Physiology, Brain Korea 21 Plus Project for Medical Science, College of Medicine, Yonsei University, Seoul, South Korea

**Keywords:** cognition, erythropoietin, inflammation, macrophage, surgery

## Abstract

Postoperative cognitive dysfunction (POCD) may be driven by transference of the innate immune response to the brain after aseptic surgical damage. Macrophages are key mediators of innate immunity that can display a pro-inflammatory M1 phenotype or an anti-inflammatory M2 phenotype. Erythropoietin (EPO) is a hematopoietic hormone that exerts anti-inflammatory effects by influencing macrophage function. We hypothesized that EPO would prevent POCD by promoting macrophage phenotype switching to the M2 phenotype post-surgery. To evaluate the effects of EPO on POCD and macrophage polarization post-surgery, we administered EPO (5,000 U/kg) with or without an arginase inhibitor (amino-6-boronohexanoic acid, 10 mg/kg) to ICR mice before and after abdominal surgery. Forty-eight hours post-surgery, we assessed memory, synapse function, and macrophage/microglial phenotypes in the spleen and hippocampus. We also investigated M1/M2 phenotypes in RAW264.7 and BV2 cells stimulated with lipopolysaccharide and interferon-γ (M1 inducers) in the presence or absence of EPO. EPO prevented POCD, decreased surgery-related synaptic dysfunction, and attenuated pro-inflammatory cytokine generation in the hippocampus. Moreover, EPO suppressed M1-related genes expression and promoted M2 genes expression in the spleen and hippocampus post-surgery. Furthermore, EPO decreased the proportions of macrophages/microglia expressing an M1 surface marker (CD40) and increased those expressing an M2 surface marker (CD206). Arginase inhibition abolished the beneficial effects of EPO on POCD. *In vitro*, EPO treatment promoted switching of RAW264.7 and BV2 cells stimulated with M1 inducers to an M2 phenotype. In conclusion, EPO prevents POCD by promoting macrophage phenotype switching toward the M2 phenotype.

## Introduction

Postoperative cognitive dysfunction (POCD) is a major neurological complication after surgery that adversely affects the quality of life, social independence, and mortality of patients (Steinmetz et al., 2009; Mashour et al., 2015). Even acute cognitive decline after surgery can result in persistent cognitive dysfunction (Saczynski et al., 2012; Inouye et al., 2016). Although several mechanisms have been associated with the development of POCD, mounting preclinical evidence suggests that inflammation in the brain (neuroinflammation) induced by systemic inflammation after surgery is a core pathogenic factor (Cibelli et al., 2010; Terrando et al., 2010; Hovens et al., 2014; Vacas et al., 2014). Furthermore, a recent meta-analysis confirmed that the systemic inflammatory response to surgery is associated with POCD in clinical settings (Peng et al., 2013).

Surgical interventions inevitably damage tissue and lead to activation of the innate immune response. Macrophage act as key mediators of innate immunity. Macrophages that are activated after surgery can infiltrate the brain parenchyma to induce microglial activation and neuroinflammation. The resulting increase in pro-inflammatory cytokine expression, especially within hippocampus, eventually leads to cognitive dysfunction (Wan et al., 2007; Dilger and Johnson, 2008; Terrando et al., 2011; Degos et al., 2013). However, macrophages can be differentially activated in response to environmental cues or pathophysiological conditions (Wang et al., 2014). Activated macrophage phenotypes include the classically activated M1 phenotype and the alternatively activated M2 phenotype. The M1 phenotype is induced by microbial products, pro-inflammatory cytokines, and destructive inflammatory mediators, such as interleukin (IL)-12, IL-23, nitric oxide, and reactive oxygen species. In contrast, the M2 phenotype can be induced by IL-4, IL-10, or IL-13, and this phenotype facilitates the clearance of cellular debris through phagocytosis, as well as the production of protective and trophic factors, such as ornithine and polyamines, through the arginase pathway. Therefore, in various disease conditions, the M1 phenotype initiates and maintains inflammation, while the M2 phenotype prevents or resolves chronic inflammation. To date, there is little evidence regarding the association between POCD and patterns of macrophage activation.

Erythropoietin (EPO) is a 30.4-kDa glycoprotein hormone that promotes red blood cell production by inhibiting the apoptosis of erythroid progenitor cells (Lombardero et al., 2011). Accordingly, EPO has been applied in clinical settings for the prevention and management of perioperative anemia (Steuber et al., 2016). The cognate receptor for EPO is expressed in various cell types; thus, EPO has pleiotropic actions in non-hematopoietic as well as hematopoietic cells (Lombardero et al., 2011). Immune cells also express the EPO receptor and are thus subject to modulation by EPO (Brines and Cerami, 2012). Several studies have reported that the treatment of activated macrophages with EPO significantly reduces the production of M1-like pro-inflammatory mediators and enhances M2-associated phagocytic activity (Nairz et al., 2011; Liu et al., 2014; Luo et al., 2016). Since EPO is commercially available in the form of recombinant human EPO (rhEPO) and widely used in the treatment of anemia now, it is very important to identify its other therapeutic potentials or mechanisms of action. Therefore, in the present study, we tested whether EPO treatment would promote the activation of macrophage/microglia toward the M2 phenotype, reduce neuroinflammation, and thus limit the extent of post-surgical cognitive dysfunction. We examined cognitive dysfunction after abdominal surgery because abdominal surgery is one of the most commonly performed surgeries and is reported to induce POCD in clinical settings (Monk et al., 2008). The model of abdominal surgery adopted in this study includes physical irritation to the viscera and mesenteric ischemia-reperfusion to mimic human exploratory abdominal surgery.

## Materials and methods

### Animals

All experimental procedures and animal care were approved by the Institutional Animal Care and Use Committee of Yonsei University, and were conducted in accordance with the Guide for the Care and Use of Laboratory Animals approved by the Association for the Assessment and Accreditation of Laboratory Animal Care. Adult male ICR mice (Orient, Seongnam, GyeongGi-Do, South Korea; aged 8–10 weeks) were used in the experiment. All mice were housed in a controlled animal facility at Yonsei University. The mice were housed in groups of five per cage on a 12-h light/dark cycle with food and water available *ad libitum*.

### Experimental design and animal grouping

The mice were randomly divided into four groups as follows: (1) control, (2) surgery, (3) EPO-control, and (4) EPO-surgery. Mice in the control group remained naïve to the experimental conditions. Mice in the EPO-control group only received rhEPO treatment, without any anesthetic or surgical procedures. Mice in the surgery group underwent abdominal surgery only. Finally, mice in the EPO-surgery group received rhEPO treatment and underwent abdominal surgery. At 1 h prior to surgery, mice in the EPO-surgery received rhEPO (5,000 U/kg, subcutaneous [s.c].; Epokine 1,000 U/ml, CJ Healthcare, Seoul, South Korea) diluted in 200 μl of phosphate-buffered saline (PBS). They received a second injection 24 h after the first injection. Mice in the EPO-control group received two injections of rhEPO with a 24 h interval between them. For surgery, the animals were anesthetized with 3% sevoflurane and maintained under anesthesia with 2% sevoflurane in a mixture of oxygen and air (inspired oxygen fraction, 0.3). Mice were placed on a heating pad while anesthetized to prevent hypothermia. Abdominal surgery was performed as previously described with some modifications (Barrientos et al., 2012; Hovens et al., 2014). Briefly, a vertical incision was made along the midline, along the linea alba, and the intestines were exteriorized and rubbed with the operator's fingers for 30 s. In addition, the superior mesenteric artery was dissected and clamped for 40 min. After removal of the clamp, the intestine was placed back into the peritoneal cavity and the abdominal muscle and skin were closed using suture. Tramadol (20 mg/kg intraperitoneal [i.p.]) was administered only to the mice undergoing surgery for post-operative analgesia.

Cognitive function was assessed using a behavioral test battery carried out 48 h post-surgery. The levels of inflammatory cytokines (IL-1β and tumor necrosis factor alpha [TNF-α]) and extracellular field potentials in the hippocampus were measured at the same time point in order to evaluate hippocampal synaptic function. To assess the pattern of macrophage/microglial activation, mRNA expression levels of selected M1-related (TNF-α and CXCL10) and M2-related (arginase-1 and IL-10) genes were measured in spleen and hippocampus tissues at 6 and 48 h post-surgery. Furthermore, proportions of M1- and M2-like macrophages/microglia were also assessed in the spleen and hippocampus tissues using flow cytometry. For the collection of spleen and hippocampal tissues, mice were anesthetized with a mixture of 30 mg/kg zoletil (Virbac Laboratory, Carros, France) and 10 mg/kg xylazine (Bayer Korea Ltd., Seoul, South Korea) and euthanized by transcardial perfusion with 0.9% normal saline at each time point. The hippocampus was isolated under a dissecting microscope as previously described (Spijker, 2011).

Finally, to confirm a beneficial role for M2 macrophage activation in cognitive function post-surgery, we performed an additional study in which a selective arginase inhibitor was co-administered with rhEPO prior to surgery. Arginase activity mediates most M2-associated anti-inflammatory effects (Pesce et al., 2009) and is positively associated with M2 macrophages (Yang and Ming, 2014). Therefore, we assessed cognitive function and levels of inflammatory cytokines in the hippocampus at 48 h post-surgery in mice that were treated with both rhEPO and an arginase inhibitor (EPO-surgery-AI group). Mice in the EPO-surgery-AI groups received rhEPO (5,000 U/kg, s.c.; Epokine 1,000 U/ml, CJ Healthcare, Seoul, South Korea) diluted in 200 μl of phosphate-buffered saline (PBS) at the same time points as the EPO-Surgery group (1 h before surgery and 24 h after the first administration), and the selective arginase inhibitor, amino-6-boronohexanoic acid (10 mg/kg, i.p.; ABH ammonium salt, Enzo Life Sciences, Farmingdale, NY, USA) dissolved in PBS 30 min before each rhEPO injection. The dose of the selective arginase inhibitor was selected based on a previous study in which 10 mg/kg of the ABH salt abolished arginase activity in M2 phenotype macrophages (Amantea et al., 2016).

### Neurobehavioral assessment

Neurobehavioral function was assessed by three different types of tests at 48 h post-surgery. Open field testing was performed to assess exploratory activity. Mice were tested in a square open field arena (40 × 40 × 40 cm) and behavior was recorded for 5 min. Total distance moved was assessed. The passive avoidance and novel object recognition tests were selected to assess hippocampal-dependent function (Antunes and Biala, 2012; Izquierdo et al., 2016). Passive avoidance testing was used to evaluate learning and memory in the mice. The apparatus used was a rectangular chamber divided into dark and lighted compartments. During the acquisition phase, mice were placed into the lighted compartment; when mice entered the dark compartment, they received an electric shock (0.5 mA) for 3 s. In the test phase, mice were place in the lighted compartment and the latency to enter the dark compartment was measured and recorded. The acquisition trial was performed at 24 h after surgery and the test trial was performed at 48 h post-surgery. Novel object recognition test was adopted to evaluate cognition, specifically recognition memory, in the mice. During the habituation phase, each mouse was allowed to explore the black box (40 × 40 × 40 cm) used for testing for 5 min. During the familiarization phase, the mice were placed into the black box, which contained two identical objects (A + A) and were allowed to explore for 5 min. During the test phase, each mouse was returned to the box with the two objects, where one object was changed to a novel object (A + B), and they were allowed to explore for 3 min. During both the familiarization and test phases, the time spent in exploring each object was measured and recorded. At the end of each test, the apparatus and objects were cleaned with 50% ethanol. The habituation phase was performed immediately prior to surgery, the familiarization phase was performed at 24 h post-surgery, and the test trial was performed at 48 h post-surgery.

All the neurobehavioral tests were recorded on video and analyzed with an image analyzing system (SMART v2.5.21 software and SMART video Tracking system, Panlab Harvard Apparatus, Barcelona, Spain) by an assessor blinded to the treatment groups. Some mice performed the open field test and the passive avoidance test sequentially. The remaining mice only performed the novel object recognition test.

### Electrophysiology

To investigate the response of the hippocampal CA1 circuit to Schaffer collateral (SC) input, hippocampal brain slices (thickness, 400 μm) were prepared from the mice at 48 h post-surgery. Briefly, the mice were anesthetized with sevoflurane and the brain was rapidly cooled via transcardial perfusion with ice-cold sucrose-artificial cerebrospinal fluid (aCSF). Brains were next removed and placed in the iced-cold sucrose-aCSF. Coronal slices were allowed to recover in aCSF for 30 min at 35°C, and then incubated in aCSF for 1–4 h at room temperature (23–25°C) before being placed into the recording chamber. Standard aCSF contained 124 mM NaCl, 2.5 mM KCl, 2.5 mM CaCl_2_, 1.3 mM MgSO_4_, 1.0 mM NaH_2_PO_4_, 26.2 mM NaH_2_CO_3_, 11 mM glucose, 2 mM Na pyruvate, and 1 mM Na ascorbate, saturated with 95% O_2_/5% CO_2_. Sucrose-aCSF contained 195.5 mM sucrose, 2.5 mM KCl, 2.5 mM CaCl_2_, 1.3 mM MgSO_4_, 1 mM NaH_2_PO_4_, 26.2 mM NaHCO_3_, 11 mM glucose, 2 mM Na pyruvate, and 1 mM Na ascorbate, saturated with 95% O_2_/5% CO_2_. All experiments were conducted at 27–29°C. For electrophysiological experiments, electrodes with 3–6 MΩ pipette resistance were used and whole-cell recordings were obtained from neurons under visual guidance using infrared-differential interference contrast optical guidance. The CA3 and dentate gyrus regions were cut away immediately prior to long-term potentiation (LTP) experiments to isolate the CA1 area. The stimuli were applied to the SC pathway and field excitatory postsynaptic potentials (fEPSPs) were recorded from the CA1 stratum radiatum using an extracellular glass pipette (3–5 MΩ) filled with aCSF. SC/commissural fibers in the stratum radiatum were stimulated using a concentric bipolar electrode placed 200–300 μm from the recording pipette. The test stimulation in all fEPSP experiments was 0.1 Hz in frequency and 0.2 ms in duration. An input/output (I/O) curve was plotted by recording fEPSPs in response to stimulation with increasing voltages (0.3–0.9 V). The stimulus for LTP was a voltage that elicited 50% of the maximum fEPSP. Baseline synaptic responses were recorded for 20 min and then LTP was induced with two high-frequency stimulation trains (100 Hz frequency, 1 s duration) separated by a 20-s inter-train interval. The recordings were performed every 30 s for 1 h using an axopatch 1D amplifier (Molecular Devices, Sunnyvale, CA, USA) digitized at 10 KHz and filtered at 2 KHz with Digidata 1322A and pClamp 9.0 software (Molecular Devices).

### Cell cultures and treatments

Mouse microglial BV2 cells were cultured in RPMI 1640 medium (Hyclone™, GE Healthcare Life Sciences, Logan, UT, USA) supplemented with 10% fetal bovine serum (FBS) and 1% penicillin-streptomycin solution. Mouse macrophage RAW 264.7 cells (KCIB, Seoul, Korea) were cultured in high-glucose Dulbecco's modified eagle medium (Hyclone™, GE Healthcare Life Sciences) supplemented with 10% FBS and 1% penicillin-streptomycin solution. Cells were incubated at 37°C in a humidified atmosphere in the presence of 5% CO_2_. For experiments, each cell line was seeded in six well plates at a density of 2 × 10^6^ per well 24 h prior to treatment. Cells were next pretreated with 5 U/ml rhEPO or PBS for 30 min and then stimulated with 50 ng/ml lipopolysaccharide (LPS) combined with 20 ng/ml interferon (IFN)-γ or PBS for 24 h.

### Enzyme-linked immunosorbent assay (ELISA)

For the *in vivo* experiment, the hippocampi were obtained at 48 h post-surgery and stored at −80°C until use. To measure levels of hippocampal IL-1β and TNF-α, the hippocampal tissues were lysed using tissue protein extraction reagent (T-PER® Tissue Protein Extraction Reagent, Thermo Scientific™, Waltham, MA, USA) containing protease and phosphatase inhibitor cocktail (100X Halt protease and phosphatase inhibitor cocktail, #1861281 Thermo Scientific™) added at a volume of 20 μl per 1 mg of tissue. The tissues were then homogenized and centrifuged at 13,000 rpm for 10 min to obtain sample supernatants. Supernatant protein concentrations were measured with a BCA Protein Assay Kit (Thermo Scientific™) according to the manufacturer specifications. Levels of mouse IL-1β and TNF-α in hippocampal lysates were assayed using high-sensitivity ELISA kits (Quantikine® ELISA, R&D Systems Inc., Minneapolis, MN, USA) according to the manufacturer specifications. Briefly, samples were added to the assay plates at a volume of 50 μl/well and incubated for 2 h at room temperature. After washing plates with the wash buffer from the kit, mouse IL-1β and TNF-α conjugates were added to each well and incubated for 2 h. The reaction was stopped and well absorbances were read at 450 nm using a microplate reader. For the *in vitro* experiment, we measured levels of IL-12 and IL-10 in BV2 and RAW 264.7 cell culture supernatants. Briefly, supernatants were collected at 24 h after LPS + IFN-γ stimulation and centrifuged at 1200 rpm for 3 min to remove cell debris. The supernatant protein concentrations were measured using a spectrophotometer (NanoDrop® ND-1000, Thermo Fisher Scientific Inc., Wilmington, DE, USA) at the A280 absorbance level. Levels of IL-12 and IL-10 in supernatants were assayed using high-sensitivity ELISA kits (Quantikine® ELISA, R&D Systems) according to the manufacturer specifications and the well absorbances were read at 450 nm using an ELISA plate reader.

### Real-time polymerase chain reaction

For the *in vivo* study, total RNA was prepared from spleen and hippocampal tissues using an RNeasy® Mini Kit (QIAGEN, Austin, Texas, USA) according to the manufacturer specifications. Briefly, recommended amounts of tissue (25 mg) were placed into the kit lysis buffer (containing an additional 10 μl β-mercaptoethanol for every 1 ml lysis buffer) and processed as recommended. For the *in vitro* study, BV2 and RAW 264.7 cells were collected at 24 h after LPS + IFN-γ stimulation and RNA was isolated using the same method as that described above. As per the manufacturer instructions, RNA was eluted with 30–50 μl of RNase-free H_2_O. Samples were immediately aliquoted and stored at −80°C until use. RNA concentrations were quantified using a spectrophotometer (NanoDrop® ND-1000) at the A260 absorbance level. The purity of the RNA was assessed by calculating the A260/A280 ratio of each sample. cDNA was synthesized from 1 μg of total RNA for each sample using the Maxime RT PreMix kit (Oligo dT Primer, iNtRON Biotechnology, Inc., Seongnam-si, Gyeonggi-do, Korea). Diluted cDNA was amplified with SYBR® Green PCR Master Mix (Applied Biosystems, Foster City, CA, USA) in a final reaction volume of 50 μl. The PCR reaction was performed using an ABI prism 7500 sequence detector (Applied Biosystems). The PCR program included initial denaturation at 95°C for 10 s followed by 40 cycles of 95°C for 5 s, 60°C for 34 s, 95°C for 15 s, 60°C for 1 min, and 95°C for 15 s. The nucleotide sequences of the primers used in this study are shown in Table 1. The cycling threshold values for TNF-α, CXCL-10, IL-1β, arginase-1, IL-10, and Ym-1 were normalized to those of β-actin.

**Table 1:** DNA sequences of the primers for reverse transcriptase polymerase chain reaction.

**Mouse cDNA**	**Primer sequences**
TNF-α	Forward, 5′ - ACGGCATGGATCTCAAAGAC - 3′
	Reverse, 5′ - AGATAGCAAATCGGCTGACG - 3′
CXCL10	Forward, 5′ - GGATGGCTGTCCTAGCTCTG - 3′
	Reverse, 5′ - TGAGCTAGGGAGGACAAGGA - 3′
IL-1β	Forward, 5′ - TGTCTTGGCCGAGGACTAAGG - 3′
	Reverse, 5′ - TGGGCTGGACTGTTTCTAATGC−3′
Arginase-1	Forward, 5′ - GAACACGGCAGTGGCTTTAAC - 3′
	Reverse, 5′ - TGCTTAGCTCTGTCTGCTTTGC - 3′
IL-10	Forward, 5′ - GCTCTTACTGACTGGCATGAG - 3′
	Reverse, 5′ - CGCAGCTCTAGGAGCATGTG - 3′
Ym-1	Forward, 5′ - GGGCATACCTTTATCCTGAG - 3′
	Reverse, 5′ - CCACTGAAGTCATCCATGTC - 3′
β-actin	Forward, 5′ - AGAGGGAAATCGTGCGTGAC - 3′
	Reverse, 5′ - CAATAGTGATGACCTGGCCGT−3′

### Flow cytometry

For fluorescent-activated cell sorting (FACS) analyses, fresh spleen and hippocampal tissues were placed on a petri dish on ice and dissociated into small pieces using a clean razor blade. FACS buffer (PBS containing 0.2% bovine serum albumin and 5 mM glucose) supplemented with 0.5 mg/mL type I collagenase was added to the dissociated tissues and the samples were incubated for 10 min. The samples were then centrifuged at 250 × g for 3 min and the resultant pellets were each resuspended in 500 μl FACS buffer. Following additional centrifugation at 250 × *g* for 3 min, the pellets were re-collected and the samples were blocked in anti-mouse CD16/CD32 (1:50, BD Biosciences, San Jose, CA, USA) for 10 min on ice. After blocking, the samples were centrifuged at 250 × g for 3 min and the resulting pellets were incubated with one of two antibody sets (M1 set: CD11b-PE, F4/80-APC, and CD40-FITC; M2 set: CD11b-FITC, F4/80-APC, CD206-PE) diluted in FACS buffer. Thereafter, the cell suspensions were filtered through a 40-μm nylon mesh cell strainer. Fluorescent cells were immediately identified using a flow cytometer (LSR II, BD bioscience, San Jose, CA, USA) and analyzed using FlowJo software version 10 (FlowJo LLC, Ashland, OR, USA). Primary gating was based on forward scatter (FSC) and side scatter (SSC) to isolate single cell events. Cell debris, dead cells, or doublets were eliminated by excluding low FSC and high SSC. Macrophage/microglia were identified using CD11b and F4/80. The M1 and M2 polarization states were identified by CD40 and CD206 expression, respectively.

### Statistical analysis

Data are expressed as the mean ± standard error of the mean (SEM). The groups were compared using a one-way analysis of variance followed by Tukey post hoc tests and unpaired *t*-tests as appropriate. Linear regression was used to estimate the slopes of the fiber volley (FV)/fEPSP ratios in the electrophysiological experiments. Differences with a *P* < 0.05 were deemed statistically significant. All statistical analyses were performed using Prism version 5.0 software (GraphPad Software Inc., La Jolla, CA, USA).

## Results

### EPO minimizes cognitive dysfunction and synaptic dysfunction following surgery

**Figure 1:**
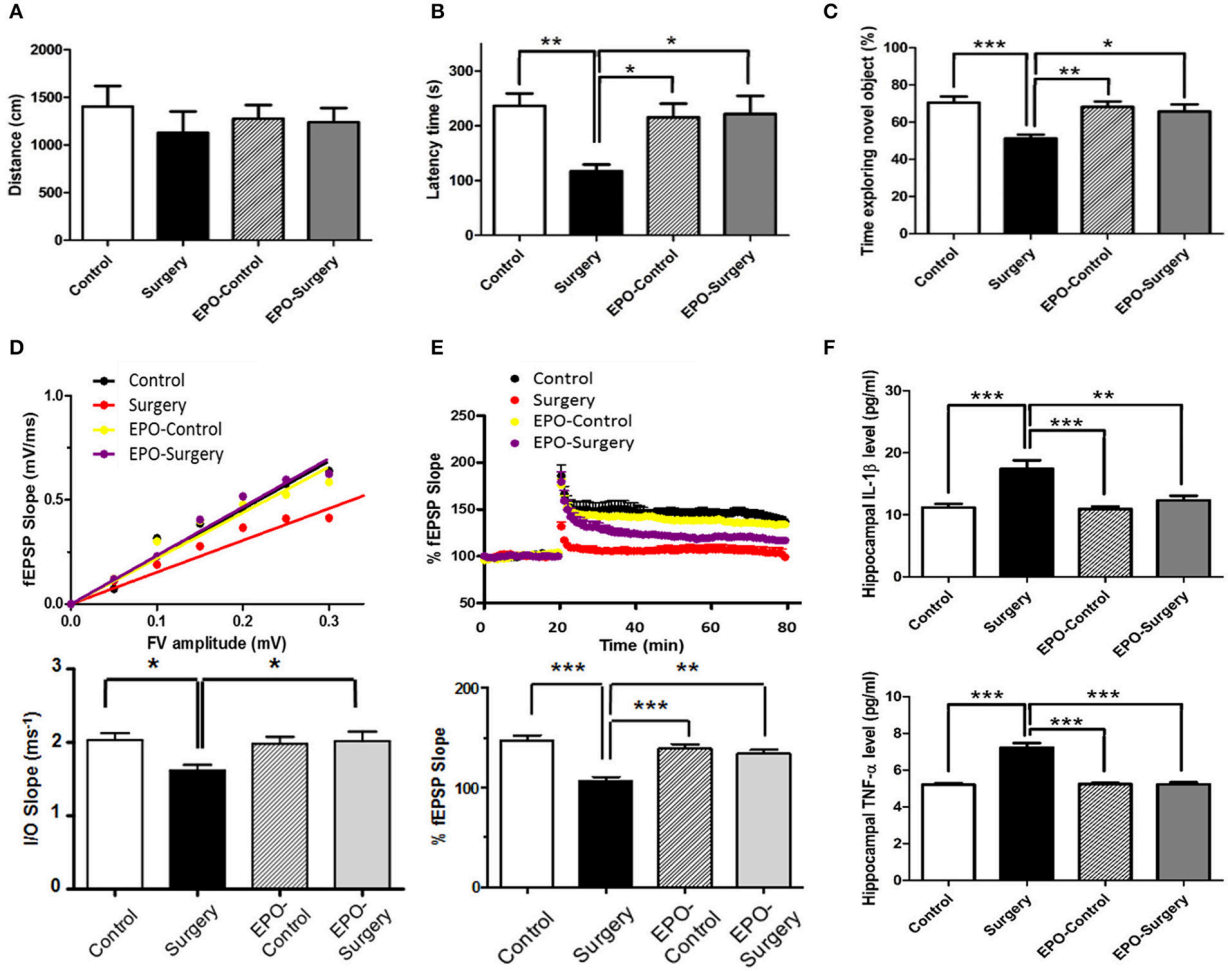
Assessments of Neurobehavioral function and hippocampal synaptic function 48 h following abdominal surgery. **(A)** Total distance moved in the open field (cm) (*n* = 11 per group). **(B)** Latency to enter the dark compartment in the test phase of the passive avoidance test (s) (*n* = 11 per group). **(C)** Proportion of time spent exploring the novel object in the test phase of the novel object recognition test (%) (*n* = 8 per group). **(D)** Upper graph, scatter plot of input/output (I/O) relationships for synaptic strength in hippocampal Schaffer collateral-CA1 synapses in each group. I/O relationships were calculated as the ratio of the fEPSP slope (between 20 and 80% of the rising part of the fEPSP) to the fiber volley amplitude. Lower graph, averaged slopes of I/O relationships for each group. **(E)** Upper graph, time course for long-term potentiation (LTP) in each group. LTP was calculated as the ratio of averaged fEPSP slopes after high frequency stimulation for 1 h to the baseline fEPSP slope before high frequency stimulation (20 min). Lower graph, averaged LTP values corresponding to the time course of LTP in the upper trace. (*n* = 6 slices from 3 mice per group). **(F)** Protein expression levels of IL-1β and TNF-α in the hippocampus (*n* = 5 per group). Control: naïve control; Surgery: abdominal surgery only; EPO-Control: erythropoietin administration only; and EPO-Surgery: erythropoietin administration and abdominal surgery. ^*^P < 0.05, ^**^P < 0.01, and ^***^P < 0.001.

With regard to the open field testing, there were no significant between-group differences in the distances the mice moved at 48 h post-surgery (Figure 1A, *P* = 0.774). For evaluations of learning and memory, there were significant between-group differences in the latency times in the passive avoidance test (Figure 1B, *P* = 0.006), and the duration spent exploring the novel object in the novel object recognition test (Figure 1C, *P* < 0.001). The latency times in the passive avoidance test were significantly shorter in the Surgery group compared to the control and EPO-surgery groups. Furthermore, mice in the surgery group spent significantly less time exploring the novel object in the novel object recognition task in comparison to all other groups. These data suggest that rhEPO prevented and/or ameliorated POCD in mice.

To investigate the effects of surgery and rhEPO on synaptic function, we measured synaptic strength and LTP in hippocampal slices using fEPSPs at 48 h post-surgery. Synaptic strength was assessed by generating I/O curves and comparing the slopes of the FV/fEPSP ratios in SC-CA1 hippocampal synapses. The synaptic strength was significantly different between the groups (Figure 1D, *P* = 0.010). The Surgery group had significantly lower fEPSP slopes at similar pre-synaptic FV amplitudes when compared with the control group, indicating a decrease in synaptic strength. The surgery-induced decrease was significantly ameliorated in the EPO-surgery group. Next, we investigated LTP in SC-CA1 synapses, which plays a key role in memory and cognitive function (Bliss and Collingridge, 1993). The differences in LTP between the groups were also statistically significant (Figure 1E, *P* < 0.001). LTP was significantly attenuated in the surgery group compared to control group. Moreover, LTP was almost completely restored in the EPO-surgery group, to a level comparable with that of the control group.

Finally, we measured the protein expression levels of IL-1β and TNF-α in hippocampal tissues (Figure 1F), since these pro-inflammatory cytokines have been reported to underlie POCD (Terrando et al., 2010; Zhang et al., 2016) and high levels of IL-1β can interfere with synaptic plasticity and LTP (Rachal Pugh et al., 2001). There were a significant between-group differences in levels of IL-1β and TNF-α in the hippocampus (IL-1β, *P* < 0.001; TNF-α, *P* < 0.001). The levels of IL-1β and TNF-α in the hippocampus were significantly higher in the surgery group compared to the control and EPO-control groups. Importantly, rhEPO administration partially attenuated the surgery-induced up-regulation of pro-inflammatory cytokines in the EPO-surgery group.

### EPO alters the expression of selected M1- and M2-related genes in spleen and hippocampal tissues following surgery

**Figure 2:**
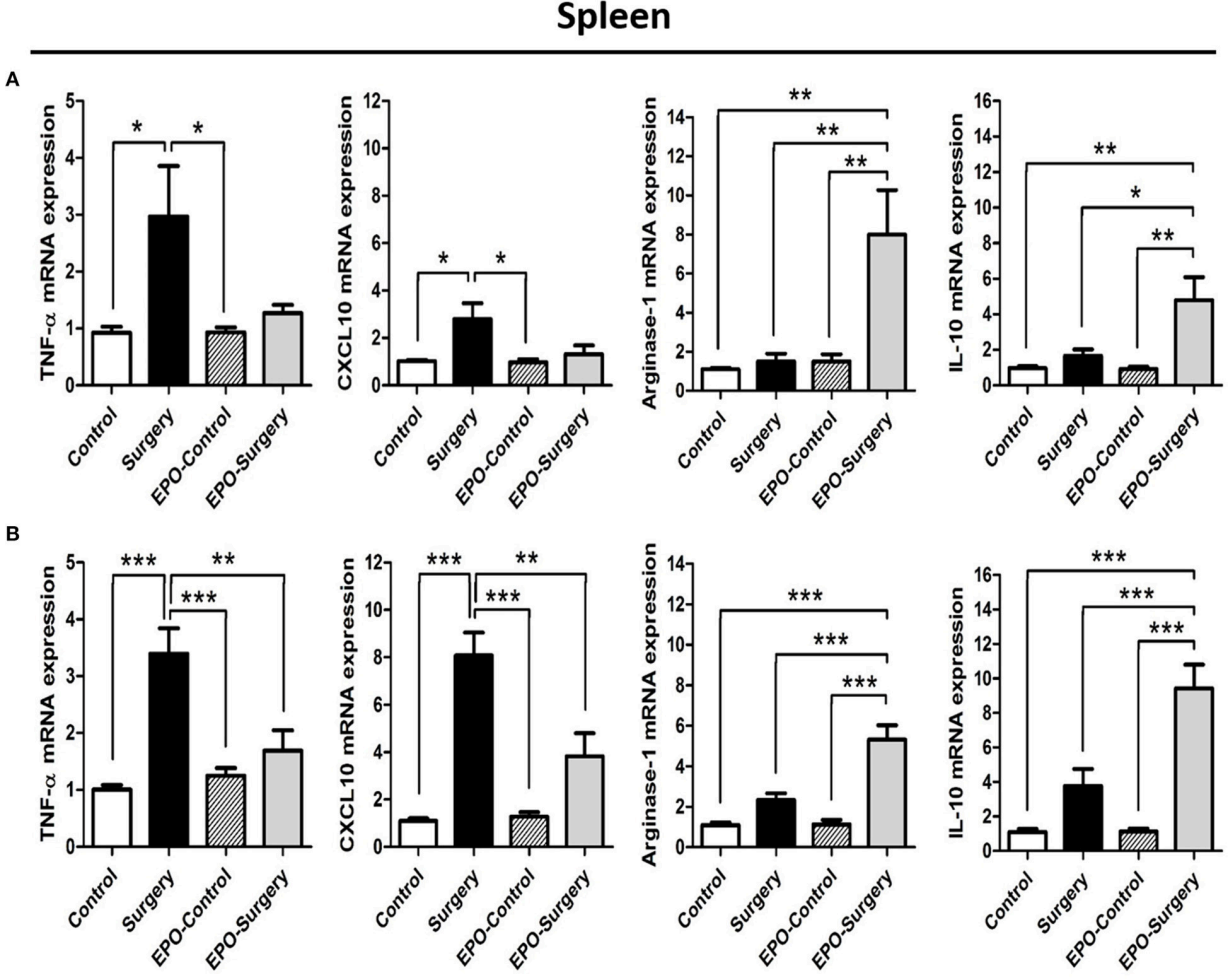
mRNA expression of selected M1-related and M2-related genes in the spleen following abdominal surgery in mice. **(A)** Expression levels 6 h post-surgery. **(B)** Expression levels 48 h post-surgery (*n* = 6 at each time point in each group). β-actin was used for normalization. ^*^P < 0.05, ^**^P < 0.01, and ^***^P < 0.001.

**Figure 3:**
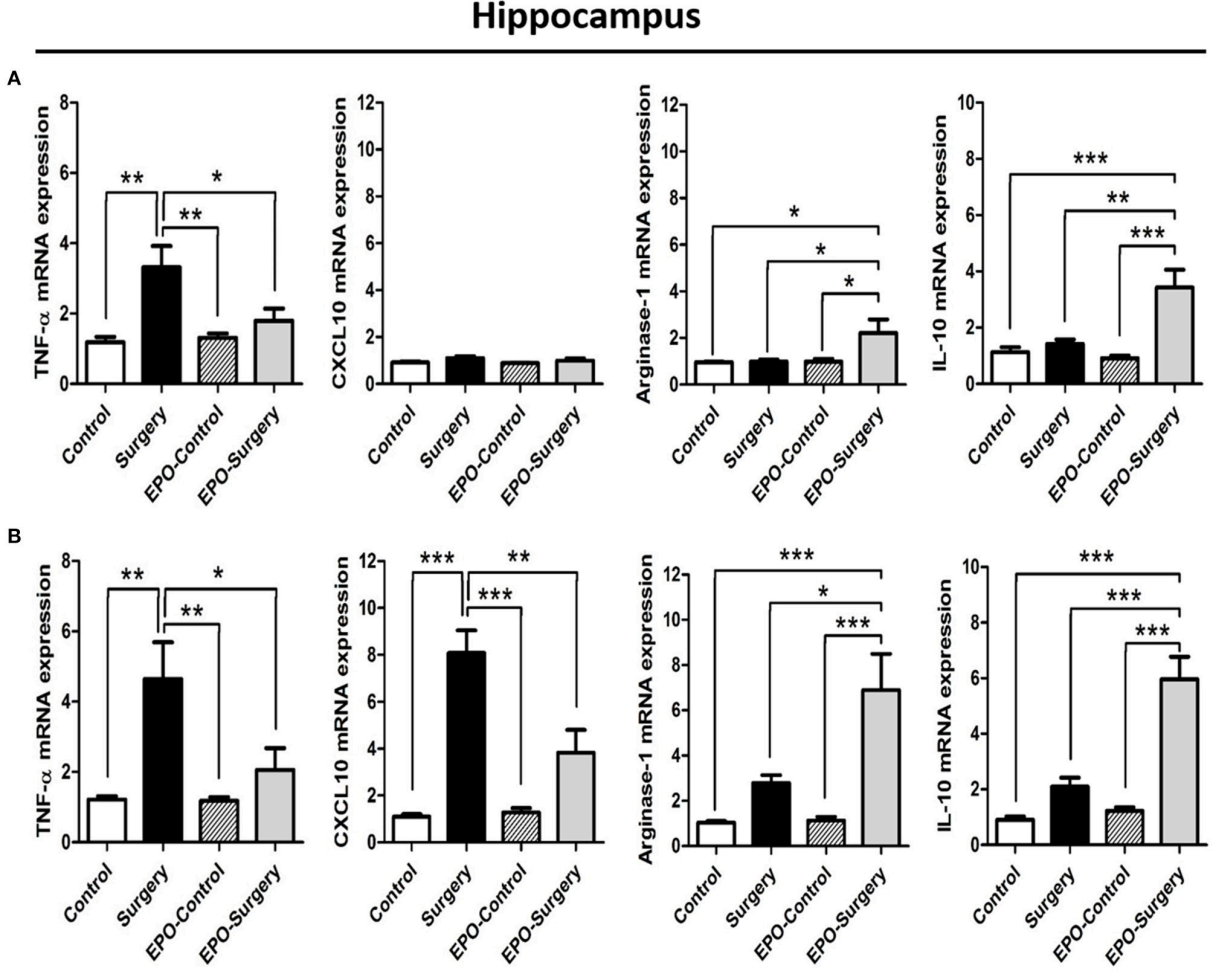
mRNA expression of selected M1-related and M2-related genes in the hippocampus following abdominal surgery in mice. **(A)** Expression levels 6 h post-surgery. **(B)** Expression levels 48 h post-surgery (*n* = 6 at each time point in each group). β-actin was used for normalization. ^*^P < 0.05, ^**^P < 0.01, and ^***^P < 0.001.

To evaluate M1 versus M2 macrophage phenotype of the mice in each group, we characterized the expression profiles of selected genes related to the M1 and M2 phenotypes in the spleen (Figure 2) and hippocampus (Figure 3) at 6 and 48 h post-surgery. There were significant differences between the groups in the expression levels of most of the selected genes (Figure 2A, spleen 6 h post-surgery: TNF-α, *P* = 0.013; CXCL10, *P* = 0.010; Arginase-1, *P* = 0.001; IL-10, *P* = 0.002; Figure 2B, spleen 48 h post-surgery: TNF-α, *P* < 0.001; CXCL10, *P* < 0.001; Arginase-1, *P* < 0.001; IL-10, *P* < 0.001; Figure 3A, hippocampus 6 h post-surgery: TNF-α, *P* = 0.002; CXCL10, *P* = 0.108; Arginase-1, *P* = 0.017; IL-10, *P* < 0.001; Figure 3B, hippocampus 48 h post-surgery: TNF-α, *P* = 0.002; CXCL10, *P* < 0.001; Arginase-1, *P* < 0.001; IL-10, *P* < 0.001). The mRNA expression of M1-related genes including TNF-α and CXCL10 was increased in the surgery group, and this increase was attenuated in the EPO-surgery group. Conversely, significant increases in M2-related gene expression including arginase-1 and IL-10 were only observed in the EPO-surgery group. Differences in M1 and M2-related gene expression among the groups were more prominent at 48 h post-surgery (Figures 2B, 3B) compared to 6 h post-surgery (Figures 2A, 3A). In addition, no significant differences in gene expression were observed between the control and EPO-control groups. Both the spleen and hippocampus demonstrated similar patterns of gene expression in each group.

### EPO increases macrophage/microglia M2 phenotype in the presence of M1-polarizing stimuli *in Vitro*

**Figure 4:**
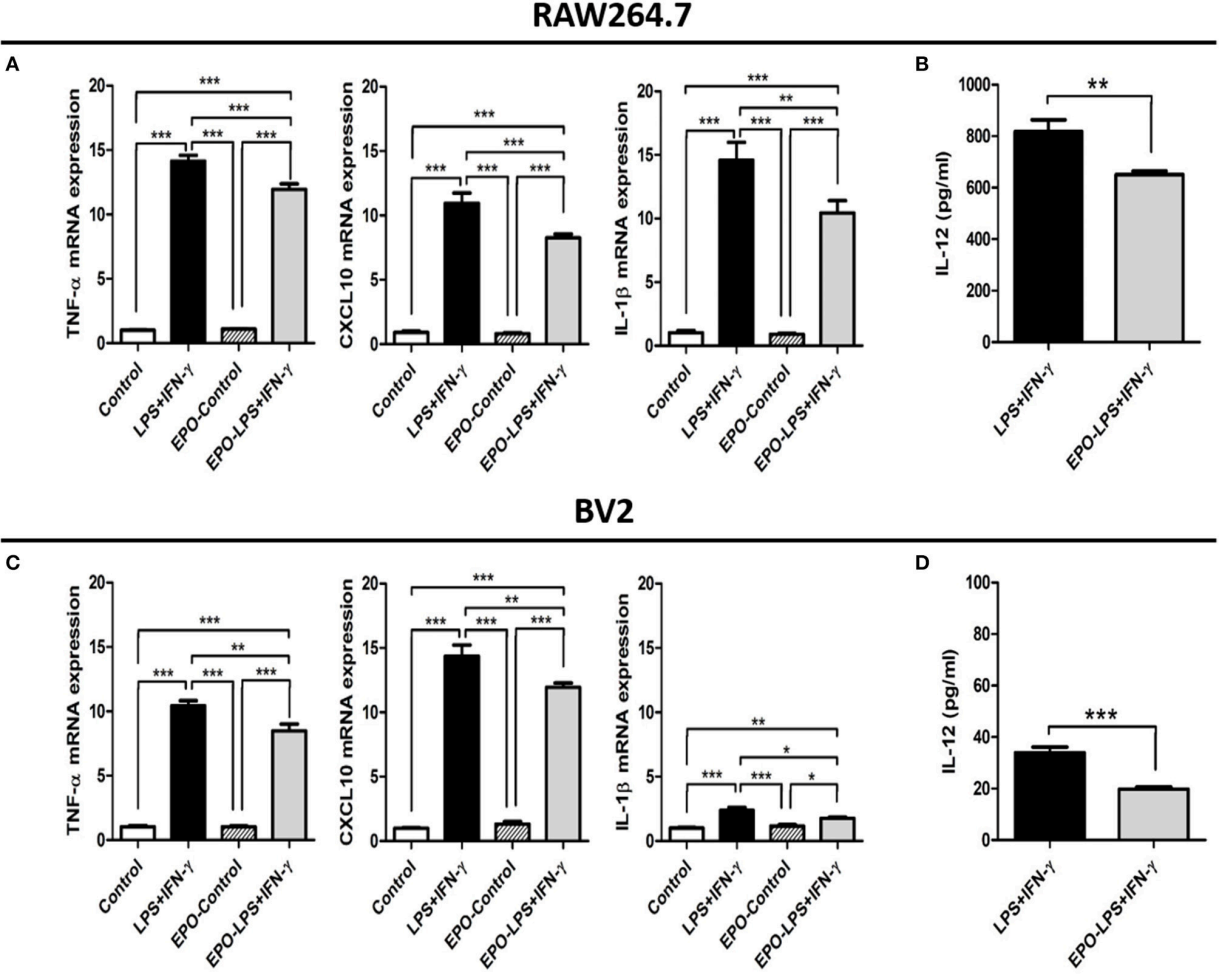
*In vitro* evaluation of the M1 phenotype in RAW 264.7 and BV2 cells after lipopolysaccharide (LPS) and interferon (IFN)-γ treatment. The mRNA expression of selected M1-related genes in RAW264.7 **(A)** and BV2 **(C)** cells. IL-12 protein expression in RAW 264.7 **(B)** and BV2 **(D)** cell supernatants. Control: non-stimulated cells; LPS+IFN-γ: M1 stimulation; EPO-Control: erythropoietin treatment only; EPO-LPS+IFN-γ: erythropoietin treatment with M1 stimulation. β-actin was used for normalization. ^*^P < 0.05, ^**^P < 0.01, and ^***^P < 0.001.

**Figure 5:**
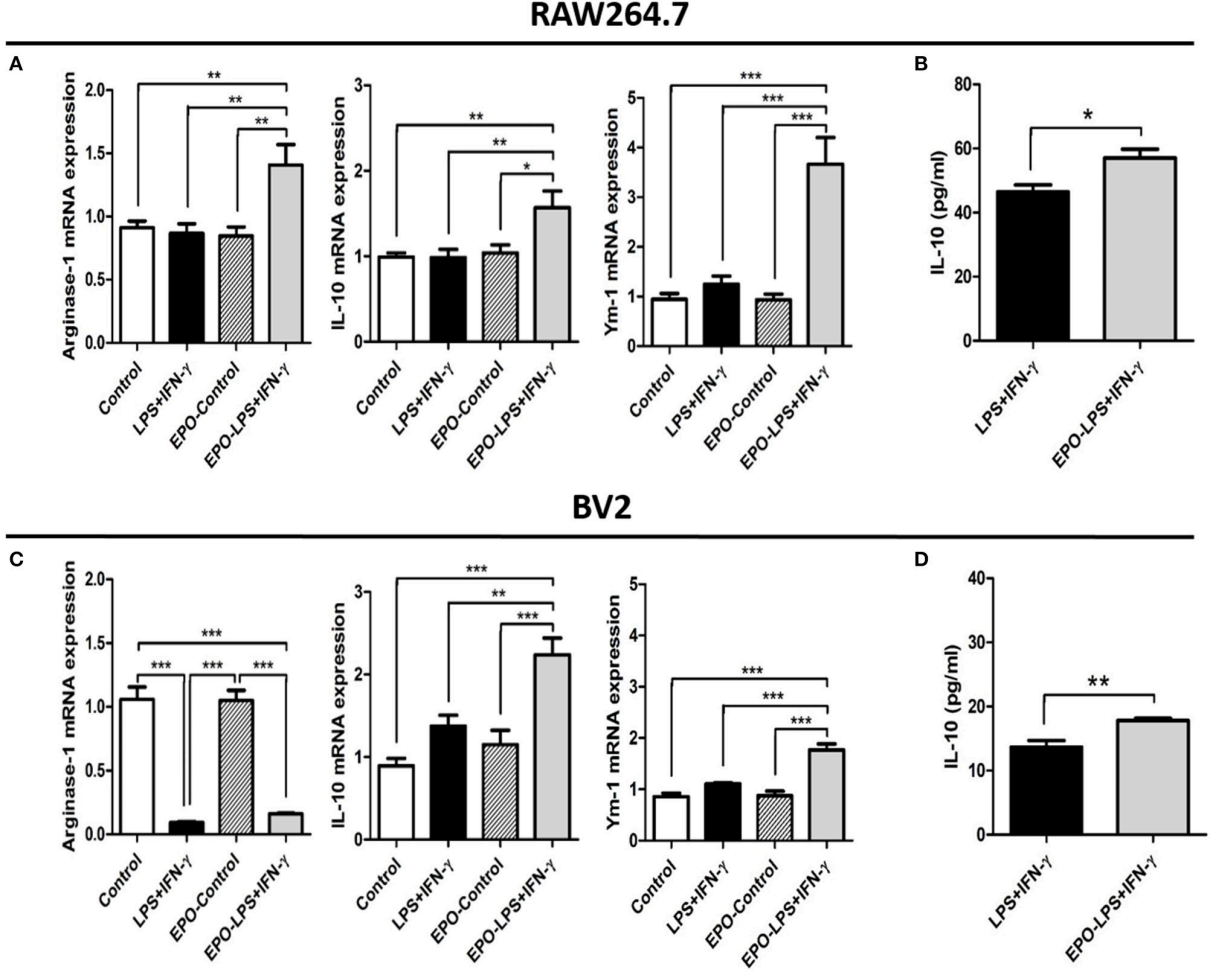
*In vitro* evaluation of the M2 phenotype in RAW 264.7 and BV2 cells after LPS+IFN-γ treatment. The mRNA expression levels of selected M2-related genes in RAW 264.7 **(A)** and BV2 **(C)** cells. IL-10 protein expression in RAW 264.7 **(B)** and BV2 **(D)** cell supernatants. β-actin was used for normalization. ^*^P < 0.05, ^**^P < 0.01, and ^***^P < 0.001.

To confirm whether rhEPO directly alters the phenotype patterns of macrophage/microglia, we assessed the effects of rhEPO on the mouse macrophage cell line RAW264.7 and the mouse microglia cell line BV2. After the stimulation with M1 inducer (LPS and IFN-γ), rhEPO, or both, there were significant differences in the mRNA expression of M1- and M2-related genes (M1: TNF-α, CXCL10, and IL-1β; M2: arginase-1, IL-10, and Ym-1) between the groups in the RAW264.7 (Figure 4A: TNF-α, *P* < 0.001; CXCL10, *P* < 0.001; IL-1β, *P* < 0.001; Figure 5A: arginase-1, *P* < 0.001; IL-10, *P* = 0.003; Ym-1, *P* < 0.001) and BV2 (Figure 4C: TNF-α, *P* < 0.001; CXCL10, *P* < 0.001; IL-1β, *P* < 0.001; Figure 5C: arginase-1, *P* < 0.001; IL-10, *P* < 0.001; Ym-1, *P* < 0.001). In both cell lines, LPS+INF-γ increased the mRNA expression of M1-related genes, and this was suppressed by treatment with rhEPO (Figures 4A,C). M2-related genes were only induced following LPS+INF-γ and treatment with rhEPO in RAW264.7 cells (Figure 5A). The patterns of gene expression for IL-10 and Ym-1 were similar in both the RAW264.7 and BV2 cell lines; however, arginase-1 gene expression was significantly suppressed by LPS+INF-γ in BV2 cells (Figure 5C). In addition, we measured secreted protein levels of IL-12 and IL-10 in the cell lines following LPS+INF-γ in the presence or absence of rhEPO treatment. After LPS+INF-γ with rhEPO treatment, IL-12 levels were significantly decreased [Figure 4B (RAW264.7), *P* = 0.008; Figure 4D (BV2), *P* < 0.001] and IL-10 was significantly increased in both cell lines [Figure 5B (RAW264.7), *P* = 0.016; Figure 5D (BV2), *P* = 0.005] compared to LPS+INF-γ alone.

### EPO alters the pattern of macrophage/microglia phenotype following surgery

**Figure 6:**
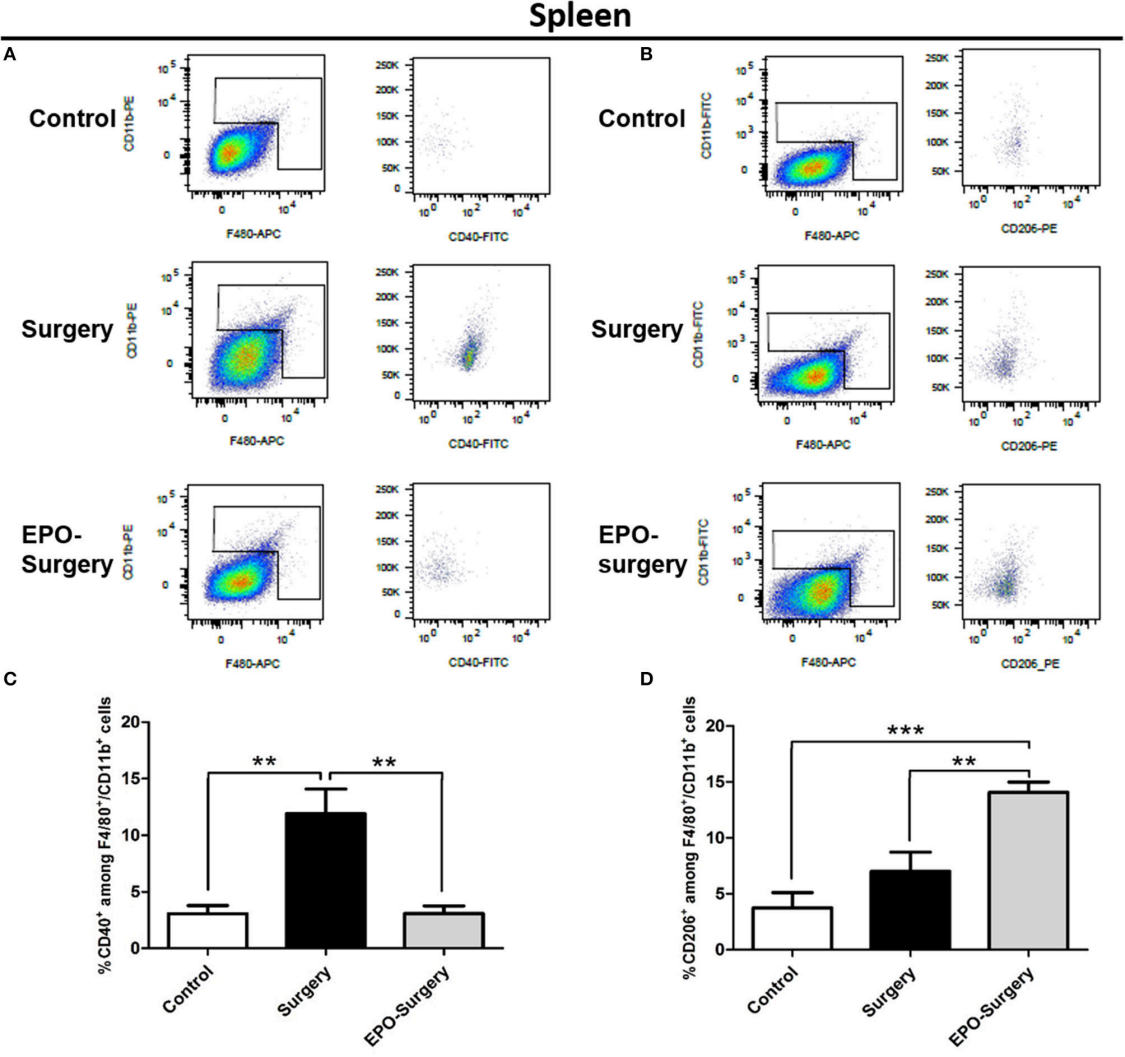
Proportions of M1 and M2 macrophages in the spleen 48 h following abdominal surgery. **(A)** Representative flow cytometry plots of F4/80^+^/CD11b^+^ cells (left row) and CD40^+^ cells (right row) in the cellular populations of each group. **(B)** Representative flow cytometry plots of F4/80^+^/CD11b^+^ cells (left row) and CD206^+^ cells (right row). **(C)** Quantification of the ratio of M1 macrophages (CD40^+^) to total macrophages (F4/80^+^/CD11b^+^). **(D)** Quantification of the ratio of M2 macrophages (CD206^+^) to total macrophages (*n* = 5 per group). ^**^P < 0.01 and ^***^P < 0.001.

**Figure 7:**
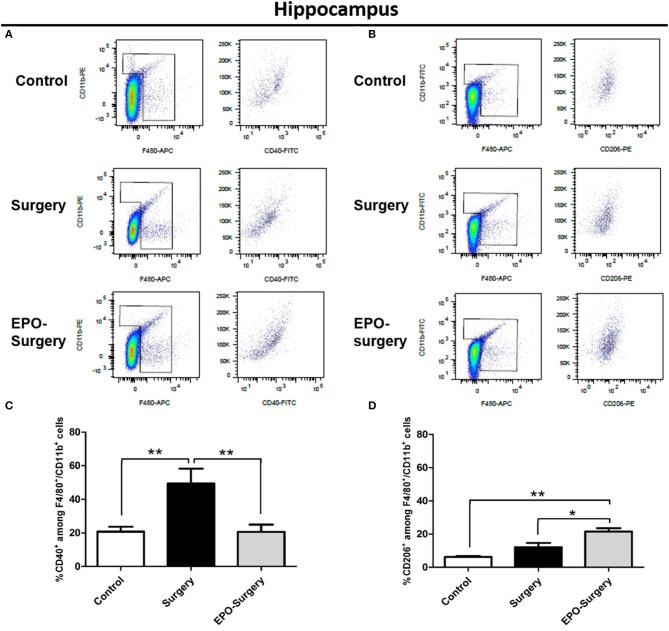
Proportions of M1 and M2 macrophage/microglia in the mouse hippocampus 48 h following abdominal surgery. **(A)** Representative flow cytometry plots of F4/80^+^/CD11b^+^ cells (left row) and CD40^+^ cells (right row) in the cellular populations of each group. **(B)** Representative flow cytometry plots of F4/80^+^/CD11b^+^ cells (left row) and CD206^+^ cells (right row). **(C)** Quantification of the ratio of M1 macrophages/microglia (CD40^+^) to total macrophages/microglia (F4/80^+^/CD11b^+^). **(D)** Quantification of the ratio of M2 macrophages/microglia (CD206^+^) to total macrophages/microglia (*n* = 6 per group). ^*^P < 0.05 and ^**^P < 0.01.

Flow cytometry was used to quantitatively assess M1 versus M2 phenotype of macrophages/microglia in the post-surgery mice. The cells were labeled with antibodies specific for macrophages/microglia (CD11b and F4/80); of these, CD40^+^ macrophages/microglia were classed as M1-phenotype and CD206^+^ macrophages/microglia were classed as M2-phenotype in the spleen (Figure 6) and the hippocampus (Figure 7). The EPO-control group was omitted from this analysis because EPO did not affect the phenotype of the resting state-macrophage/microglia as shown previously (Figures 2–5). The relative proportions of CD40^+^ cells and CD206^+^ cells among the CD11b^+^/F4/80^+^ cells were compared, and significant differences were found between the groups (Figure 6C: CD40^+^ cells in the spleen, P < 0.001; Figure 6D: CD206^+^ cells in the spleen, *P* < 0.001; Figure 7C: CD40^+^ cells in the hippocampus, *P* = 0.005; Figure 7D: CD206^+^ cells in the hippocampus, *P* < 0.001). As depicted in Figure 6C, the proportion of CD40^+^ cells in the spleen was significantly increased in the surgery group compared to the control group, whereas an increased proportion of CD40^+^ cells was not observed in the EPO-surgery group. Conversely, there were no significant differences in the proportion of CD206^+^ cells between the surgery and control groups; however, the proportion of CD206^+^ cells was significantly greater in the EPO-surgery group compared to the control and surgery groups (Figure 6D). Similar patterns of polarization were observed in the hippocampus compared to the spleen (Figures 7C,D).

### Arginase inhibition abolishes EPO-induced improvements in POCD

**Figure 8:**
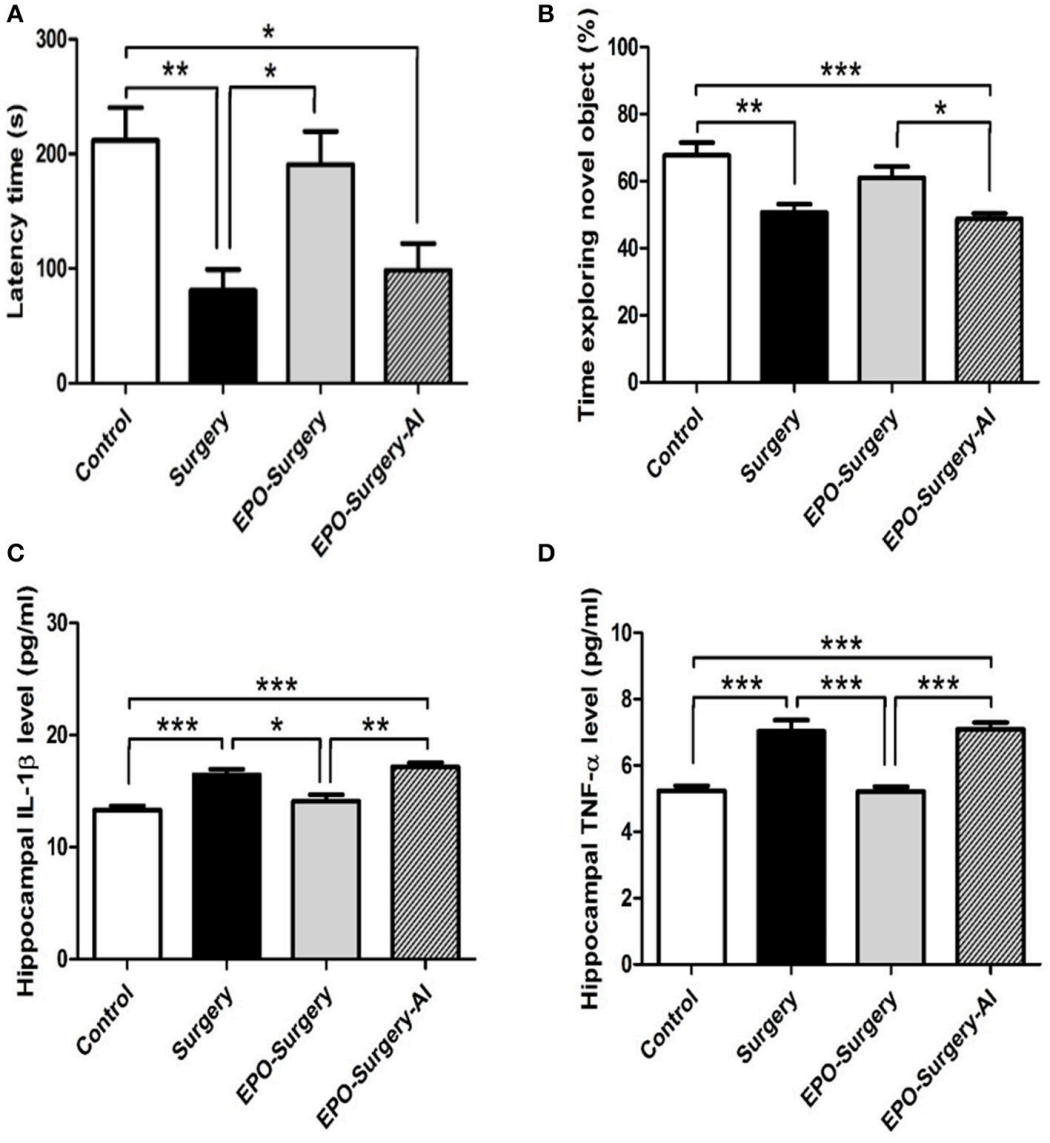
Neurobehavioral functional assessments and hippocampal pro-inflammatory cytokine expression after arginase inhibitor administration. The EPO-Surgery-AI group received a selective arginase inhibitor prior to each erythropoietin treatment, and received abdominal surgery. **(A)** Latency to enter the dark compartment in the test phase of the passive avoidance test (s). **(B)** Proportion of time spent exploring the novel object in the test phase of the novel object recognition test (%) [*n* = 8 per group in **(A,B)**]. **(C)** IL-1β and **(D)** TNF-α protein expression in the hippocampus [*n* = 5 per group in **(C,D)**]. ^*^P < 0.05, ^**^P < 0.01, and ^***^P < 0.001.

Arginase is thought to mediate the anti-inflammatory effects of M2 macrophages (Pesce et al., 2009). Therefore, we assessed post-operative cognitive function and inflammatory cytokine levels in the hippocampus following administration of a specific arginase inhibitor with EPO in order to estimate the influence of macrophage M2 polarization on POCD. There were significant between-group differences in the results from passive avoidance and novel object recognition tests (Figure 8A: passive avoidance test, *P* = 0.001; Figure 8B: novel object recognition test, *P* < 0.001). As we previously observed in the passive avoidance test, the latency to enter the dark box of the surgery group was significantly shorter than that of the control group, whereas the latency in the EPO-surgery group was similar to that of the control group. Arginase inhibition prevented the restorative effect of EPO on the latency, such that the latency of the EPO-surgery-AI group was similar to that of the surgery group. In the novel object recognition test, the time spent exploring the novel object was close to 50% in the EPO-surgery-AI group, suggesting that these mice had trouble in distinguishing the novel object, and thus impaired cognitive function. Furthermore, the levels of the pro-inflammatory cytokines IL-1β and TNF-α in the hippocampus were significantly different between the groups (Figure 8C: IL-1β, *P* < 0.001; Figure 8D: TNF-α, *P* < 0.001). The levels were higher in the EPO-surgery-AI group than in the control and EPO-surgery groups.

## Discussion

The main findings of this study are as follows: (1) EPO treatment suppressed increases in pro-inflammatory cytokines in the hippocampus following surgery, and appeared to prevent or alleviate the post-operative impairments in LTP and synaptic dysfunction, which was associated with decreased POCD. (2) EPO increased the proportion of macrophages/microglia with an M2-phenotype *in vivo* in spleen and hippocampal tissues post-surgery and *in vitro* after stimulation of M1 inducers. (3) Arginase inhibition abolished EPO-associated improvements in cognitive function and neuroinflammation post-surgery.

The core pathophysiology of POCD has been reported to involve neuroinflammation that results from systemic inflammation after surgical damage (Degos et al., 2013; Vacas et al., 2014). Elevated levels of pro-inflammatory cytokines such as IL-1β in the hippocampus can impair synaptic function and lead to cognitive dysfunction (Rachal Pugh et al., 2001; Cibelli et al., 2010; Terrando et al., 2010; Zhang et al., 2016). In our study, EPO improved the behavioral indicators of POCD, and prevented the increase in pro-inflammatory cytokine expression in the hippocampus post-surgery. Although previous evidence indicates that EPO has anti-inflammatory effects in the central nervous system and on the periphery (Agnello et al., 2002; Nairz et al., 2011), the present study is the first to demonstrate that the anti-inflammatory effects of EPO may be attributable to M2-phenotype macrophage. Specifically, we found that EPO increased the expression of M2-related genes and increased the proportion of M2-like macrophages in the spleen and hippocampus post-surgery, and our *in vitro* experiment demonstrated that EPO could directly change the phenotype of activated macrophage. Furthermore, the effects of EPO on cognitive function and neuroinflammation were abolished by the inhibition of arginase, which is thought to mediate M2 anti-inflammatory effects. Macrophages can be characterized as M1 or M2 according to the metabolism of arginine (Rath et al., 2014; Yang and Ming, 2014). While M1 macrophages metabolize arginine via inducible nitric oxide synthase to produce the cytotoxic radical nitric oxide, M2 macrophages utilize arginase to metabolize arginine to ornithines and polyamines that promote cell proliferation and tissue repair. Therefore, it may be inferred that the effect of EPO on macrophage phenotype led to the suppression of pro-inflammatory cytokine expression in the hippocampus and thus prevented POCD. Additionally, our *in vitro* results indicate that EPO did not induce an M2 phenotype, but rather induced M2 switching in the presence of M1 activation cues. We did not observe any changes in M1 or M2-related gene expression when cells were treated with EPO in the absence of M1 inducers. Consistent with this idea, EPO did not alter the expression of any M1- or M2-related genes in the absence of a surgical insult *in vivo*.

EPO receptors on erythroid cells are comprised of two identical EPO receptor subunits, whereas non-erythroid cells such as immune cells express a heterodimeric receptor that consists of an EPO receptor subunit and a β common receptor subunit (Brines and Cerami, 2012). Thus, EPO receptors in erythroid tissues have a much higher affinity for EPO than those in other tissues. Indeed, the activation of non-hematopoietic EPO receptors may require a higher dose of EPO than that used to stimulate hematopoietic EPO receptors. For example, 600 U/kg EPO is sufficient to reduce the need for perioperative allogenic blood transfusion in patients (Steuber et al., 2016; Zhao et al., 2016). In contrast, Nairz et al. demonstrated that 5,000 U/kg EPO in mice influenced the actions of activated macrophages through the EPO receptor (Nairz et al., 2011). For this reason, we used a dose of 5,000 U/kg EPO in our experiment to test the utility of EPO for altering macrophage polarization in POCD. One limitation of this approach is that high doses of EPO are associated with an increased risk of thrombosis in clinical settings (Bohlius et al., 2006; Corwin et al., 2007; van der Meer and van Veldhuisen, 2011). Alternative compounds such as carbamylated EPO are currently being developed to circumvent such side effects. Carbamylated EPO is an EPO receptor agonist that lacks erythropoietic and hematopoietic activities (Brines and Cerami, 2012), which may make it safer for controlling the postoperative innate immune response in patients. Carbamylated EPO was recently tested in several disease models and showed promising results (van Rijt et al., 2014; Chen et al., 2015).

Although little is known about how macrophage polarization affects postoperative recovery, considerable insight can be derived from studies of infection and cancer (Labonte et al., 2014). M1 macrophage polarization is largely related to protection against bacterial infection and cancer based on the bactericidal and anti-tumoral effects of M1 macrophages. Alternatively, viral infection more commonly leads to M1 polarization and the severity of inflammation is often correlated with disease severity. Therefore, M2 polarization may play a role in alleviating the severity of viral infection. Expanding on this idea, our study suggests that M2 polarization alleviates surgery-induced aseptic inflammation to prevent POCD.

This study had some limitations. First, although the trajectory of cognitive function should be evaluated over a period that includes complete surgical recovery in order to properly assess POCD (Nadelson et al., 2014), we assessed cognitive function in mice at a single time point 2 days after abdominal surgery. This time point was selected based on the results of a previous study using a similar surgery model that reported that cognitive impairments were most pronounced during the first week post-surgery (Hovens et al., 2014). Second, we were not able to distinguish between the effects of EPO on microglia versus macrophages in our *in vivo* experiment; blood-brain barrier permeability can be increased with surgery (Bi et al., 2017; Yang et al., 2017), and systemically administered EPO has been reported (observed?) to cross the blood-brain barrier not only in some brain injury models (Brines et al., 2000; Sirén et al., 2001) but also in healthy mice (Ehrenreich et al., 2004). However, a previous study indicated that the infiltration of systemic macrophages into the hippocampus is essential for the development of POCD (Degos et al., 2013), suggesting that macrophages might be largely responsible for the effects of EPO in POCD. Moreover, the direct stimulation of microglia with EPO *in vitro* produced a different pattern in arginase expression from that observed in the hippocampus. Third, we could not exclude the possibility that EPO affects macrophage/microglia partly through the actions on the other cell types *in vivo* since various cell types have EPO receptors. Fourth, we did not include a group that only received anesthesia in this study, which may be a weakness of the study since anesthesia has been reported to contribute to POCD (Arora et al., 2014). Finally, narcotic analgesics are known risk factors of postoperative cognitive dysfunction (Bilotta et al., 2013), and therefore the effect of tramadol given to the mice undergoing surgery cannot be completely excluded.

## Conclusion

Our data support the idea that EPO shifts macrophage activation from an M1 to an M2 phenotype, and this may be associated with its ability to prevent POCD.

## Author contributions

JL and B-NK conceived and designed the experiments. JL, EHK, SK, SYC, and J-HJ performed the experiments. JL and B-NK analyzed the data and wrote the paper. EJK and SC provided experimental technical support and contributed to the paper revision. All authors read and approved the final manuscript.

### Conflict of interest statement

The authors declare that the research was conducted in the absence of any commercial or financial relationships that could be construed as a potential conflict of interest.
